# Bioactive Candy: Effects of Licorice on the Cardiovascular System

**DOI:** 10.3390/foods8100495

**Published:** 2019-10-14

**Authors:** Mikkel R. Deutch, Daniela Grimm, Markus Wehland, Manfred Infanger, Marcus Krüger

**Affiliations:** 1 Department of Biomedicine, Aarhus University, 8000 Aarhus C, Denmark; mikkelrd@gmail.com (M.R.D.); dgg@biomed.au.dk (D.G.); 2 Clinic for Plastic, Aesthetic and Hand Surgery, Otto von Guericke University, 39120 Magdeburg, Germany; markus.wehland@med.ovgu.de (M.W.); manfred.infanger@med.ovgu.de (M.I.); 3 Gravitational Biology and Translational Regenerative Medicine, Faculty of Medicine and Mechanical Engineering, Otto von Guericke University, 39120 Magdeburg, Germany

**Keywords:** licorice, glycyrrhizin, glycyrrhetinic acid, glabridin, 11-β-dehydrogenase isozyme 2, hyperaldosteronism, hypokalemia, hypertension

## Abstract

Licorice, today chiefly utilized as a flavoring additive in tea, tobacco and candy, is one of the oldest used herbs for medicinal purposes and consists of up to 300 active compounds. The main active constituent of licorice is the prodrug glycyrrhizin, which is successively converted to 3β-monoglucuronyl-18β-glycyrrhetinic acid (3MGA) and 18β-glycyrrhetinic acid (GA) in the intestines. Despite many reported health benefits, 3MGA and GA inhibit the 11-β-hydrogenase type II enzyme (11β-HSD2) oxidizing cortisol to cortisone. Through activation of mineralocorticoid receptors, high cortisol levels induce a mild form of apparent mineralocorticoid excess in the kidney and increase systemic vascular resistance. Continuous inhibition of 11β-HSD2 related to excess licorice consumption will create a state of hypernatremia, hypokalemia and increased fluid volume, which can cause serious life-threatening complications especially in patients already suffering from cardiovascular diseases. Two recent meta-analyses of 18 and 26 studies investigating the correlation between licorice intake and blood pressure revealed statistically significant increases both in systolic (5.45 mmHg) and in diastolic blood pressure (3.19/1.74 mmHg). This review summarizes and evaluates current literature about the acute and chronic effects of licorice ingestion on the cardiovascular system with special focus on blood pressure. Starting from the molecular actions of licorice (metabolites) inside the cells, it describes how licorice intake is affecting the human body and shows the boundaries between the health benefits of licorice and possible harmful effects.

## 1. The Sweet “Father of Herbal Medicine”

**Figure 1:**
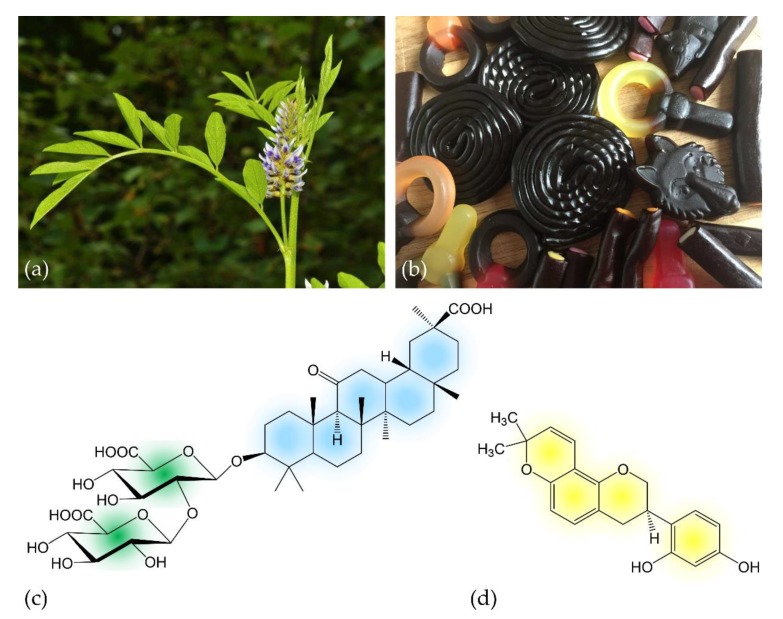
(**a**) Inflorescence of *Glycyrrhiza glabra* L.; (**b**) licorice-containing candies; (**c**) chemical structure of the prodrug glycyrrhizin (C_42_H_62_O_16_), the main active compound of licorice. The molecule consists of two molecules of glucuronic acid (left) that are linked to 18β-glycyrrhetinic acid; (**d**) chemical structure of glabridin (C_20_H_20_O_4_), a further bioactive licorice compound. Colors indicate molecule structures used in following schematics.

Licorice is the root of the legume *Glycyrrhiza glabra* (Figure 1a) that grows in varieties in warm areas like the Middle East, Asia and Southern Europe. It is one of the oldest used herbs in ancient medicine and referred to as “the father of herbal medicine” [1]. Licorice, from which a sweet flavor can be extracted, has been used in herbal and traditional medicine in both Eastern and Western cultures dating back to beyond 4000 BC [2]. The early Egyptians and Assyrians are known to have cultivated the ‘sweet root’ that was later imported to China, where it has been used for centuries under the name ‘*Gan Cao*’ [3]. It has also been described by ancient Greeks, including Hippocrates and Theophrastus, as well as by Romans [2,4]. Today, the Scandinavian countries seem to have the most consumers of licorice; however, licorice intake is also a popular strategy to quench thirst during Ramadan (based on its historical utilization in the desert or on battlefields, where travelers and soldiers drank licorice extracts to combat thirst sensation on long marches). Although the main active compound glycyrrhizin is considered to be 50-times sweeter than sucrose [5], licorice is rarely used for sweetening purposes alone due to its associated flavor and the brownish color that would be imparted to non-acidic foods [2,6]. Since the 18th century, the primary use comprises mainly licorice extracts (in pharmacy called *Succus liquiritae*) as a flavoring additive in tea, tobacco, candy (Figure 1b) and other sweets, but the licorice root itself (*Liquiritae radix*) is still used as a dietary supplement in some parts of the world [7]. Among people preferring alternative or complementary medicine, historical uses for licorice were revived and are still practiced today [8,9,10].

On the one hand, the health benefits ascribed to licorice are numerous: for centuries it has been used in herbal and folk medicine to treat multiple diseases such as gastrointestinal symptoms and respiratory diseases [10]. The broad spectrum of activities known today comprises immunostimulatory and anti-ulcer effects [11,12,13], anti-viral and anti-microbial effects [14,15], hepatoprotective [16,17], anti-carcinogenic [18] and several other positive effects that contribute to the protection of the nervous, respiratory, endocrine and cardiovascular system [9]. Licorice is also effective against gastrointestinal problems by repairing the inner layer of the stomach and cleansing the respiratory system by increasing the production of mucus. Furthermore, other extracts of the licorice root have been tested in the treatment of gastritis induced by *Helicobacter pylori* and showed promising results [19,20]. The polyphenolic flavonoid glabridin possesses hypoglycemic effects by modulating glucose and lipid metabolism [21,22], similar to effects attributed to green tea extract [23]. On the other hand, it is well-known that consuming excessive quantities of licorice can impact upon cardiometabolic health by elevating blood pressure (BP), and thus, may be a cause of hypertension and other cardiovascular complications [24,25,26,27,28,29,30].

Hypertension is one of the major concerns for our healthcare system and was the leading contributor to premature death in 2015 [31]. Due to the higher arterial BP, it has been proven to be a major risk factor of cardiovascular diseases (CVD). The global prevalence of hypertension was estimated to be about 1.13 billion. Generally, hypertension is the cut-off BP value, where the benefits of treatment outweigh the associated risks. According to the European Society of Cardiology (ESC) “hypertension” is defined as a systolic BP ≥ 140 mmHg and a diastolic BP ≥ 90 mmHg [32]. Hypertension is divided into primary and secondary forms. It is a multifactorial disease, where the contribution of different factors is variable and with a small effect size. Most of the patients have no clear etiology, and they are classified as having primary hypertension. According to Charles et al. [33], about 5–10% of hypertensive patients have secondary hypertension, a result of a different disease affecting the cardiovascular system, such as renal diseases, primary hyperaldosteronism or obstructive sleep apnea.

Licorice and other drugs affecting the hormonal regulation of the water and electrolyte balance could be causing primary hypertension. To examine the actual cause of hypertension, some tests are needed. This would include measurements of plasma aldosterone and plasma renin. Aside from licorice, plenty of additional risk factors increase the possibility of developing hypertension [32].

In this review, we summarized and evaluated current literature about the effects of licorice ingestion on the cardiovascular system with special focus on BP. The literature was primarily identified using online databases. The search was completed on 24/9/2019. The primary registers included PubMed, Embase and ClinicalTrials.gov. Keywords that were used in the search included both “licorice” and “liquorice”. Both variations were used to ensure a more complete search, since “licorice” is widely used in American literature whereas “liquorice” is common in British literature. In PubMed, the search for “liquorice” alone gave 4347 results, while “liquorice and hypertension” narrowed it down to 364 results. “Liquorice and cardiovascular disease” gave 379 results; “*Glycyrrhiza* and hypertension” resulted in 255 hits. We thoroughly collected information about the molecular and physiological mechanisms of licorice in order to explore the effects and prevalence of licorice intake in general. This way, we want to show the boundaries between its health benefits and possible harmful effects.

## 2. Pharmacological Effects of Licorice

### 2.1. Licorice Digestion and Chemistry of Metabolites

**Figure 2:**
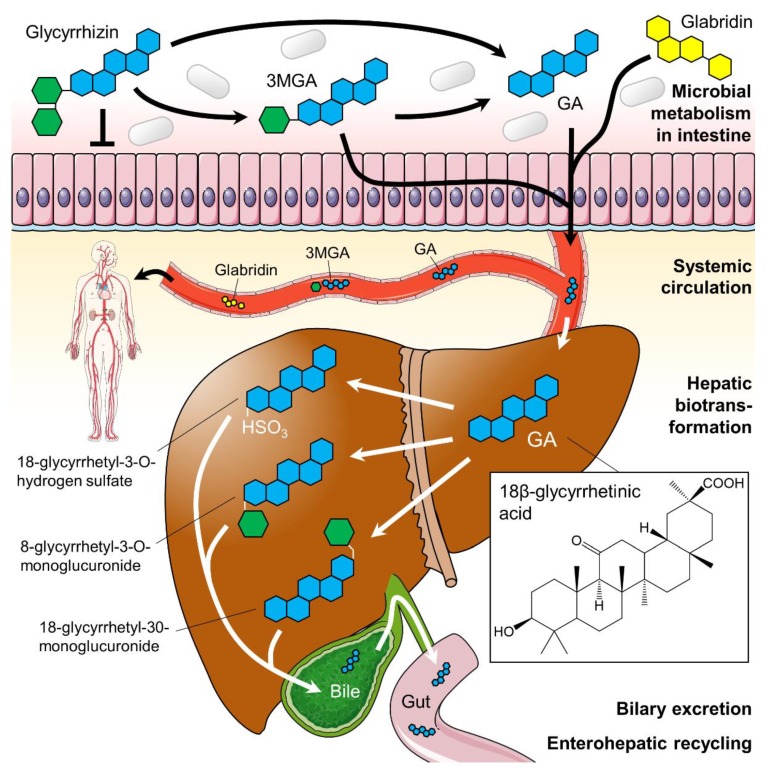
Suggested glycyrrhizin metabolism. Dependent on the gut microbiome glycyrrhizin is stepwise hydrolyzed to 3β-monoglucuronyl-18β-glycyrrhetinic acid (3MGA) and 18β-glycyrrhetinic acid (GA; blue structure) in the intestines. Both 3MGA and GA were absorbed from the gut and transported systemically in the bloodstream. In the liver, they undergo hepatic biotransformation before products were excreted via bile. The flavonoid glabridin (yellow structure) is also absorbed from the gut and circulates in the blood in its aglycone form. The hepatic metabolization of glabridin is not shown here. Green hexagons: glucuronic acid. Parts of the figure were drawn by using pictures from Servier Medical Art (http://smart.servier.com), licensed under a Creative Commons Attribution 3.0 Unported License (https://creativecommons.org/licenses/by/3.0).

Licorice consists of up to 300 active compounds comprising phenolic acids, flavonoids, flavans, chalcones, isoflavans (including glabridin, the main compound found in the hydrophobic fraction of licorice extract) and isoflavonoids [10]. A species-dependent content of 3 to 5% the triterpenoid saponin glycyrrhizin (Figure 1c) accounts for the sweet taste of licorice root and is the main active constituent of licorice [6,34]. Although the presence of glycyrrhizin in licorice has been known for over 200 years, detailed chemical investigations have not been conducted until the mid of the 20th century [35]. In the licorice root, tribasic glycyrrhizin naturally occurs in form of its calcium and potassium salts. After oral ingestion, glycyrrhizin (which itself possesses only poor oral bioavailability) is successively hydrolyzed to 3β-monoglucuronyl-18β-glycyrrhetinic acid (3MGA) and the aglycone 18β-glycyrrhetinic acid (GA; also known as enoxolone) by intestinal bacteria possessing specialized β-glucuronidases [36,37]. GA is often considered as the active metabolite of licorice [38,39,40], but its pharmacokinetics seem to be more complex. After rapid absorption from the gut, 3MGA and GA circulate in the bloodstream. From there, they are transported to the liver by carrier molecules, where they are metabolized (Figure 2). In humans, hepatic processing is not yet clearly defined, but it is apparent that each metabolite can undergo further conjugation or reduction followed by biliary excretion [6]. The products are likely re-metabolized by the gut microbiome and thereby subjected to enterohepatic recycling requiring several days for complete elimination [41].

The further bioactive constituent, glabridin (Figure 1d), has shown low oral bioavailability in rats. Microsomal studies by Cao et al. [42] demonstrated that glabridin is mainly metabolized by hepatic glucuronidation. They also found that the intestine contributes to glabridin glucuronidation to a much lesser extent. After the intestinal absorption process involving P-glycoprotein, glabridin appears in the human plasma and in the liver as the free (aglycone) form that also circulates within the bloodstream [43,44].

The digestion of licorice is still not completely understood. Interestingly, the bioavailability of glycyrrhizin is reduced when consumed as licorice [45], suggesting that some components of the licorice root may interact with glycyrrhizin during intestinal absorption, reducing its oral bioavailability [46]. Some recent animal studies on rats indicated that there might be further metabolites of GA as causal candidates for the described pharmacological effects [47,48]. In addition, it should be mentioned that the enterohepatic circulation of GA has not yet been studied in humans. However, similar steps can be expected, because GA metabolites can be hydrolyzed by human gastrointestinal bacteria as well [6].

### 2.2. Pharmacodynamics of Licorice Constituents and Metabolites

**Figure 3:**
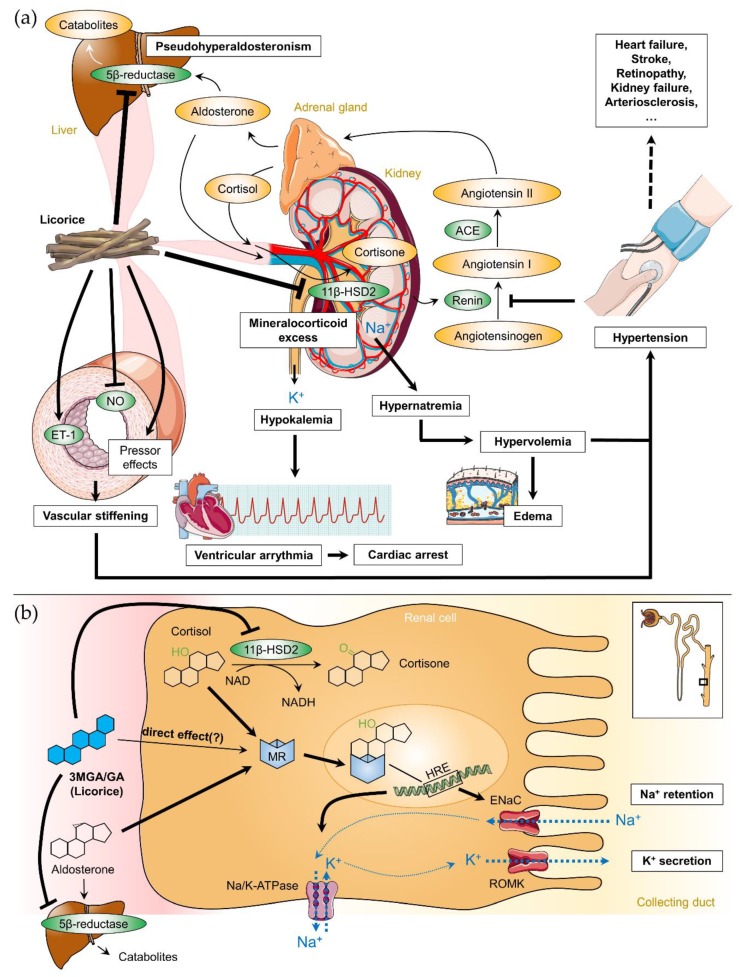
(**a**) Correlation between licorice intake, the renin-angiotensin-aldosterone-system and licorice-induced adverse effects on the cardiovascular system. (**b**) Detailed pharmacodynamics of 3β-monoglucuronyl-18β-glycyrrhetinic acid (3MGA) and 18β-glycyrrhetinic acid (GA; blue structure) in the kidney. In addition to a possible direct binding to the mineralocorticoid receptor (MR), 3MGA and GA have inhibiting effects on 11β-hydroxysteroid dehydrogenase type 2 (11β-HSD2) and 5β-reductase. 11β-HSD2 converts cortisol to cortisone; 5β-reductase is involved in the degradation of aldosterone in the liver. Inhibition of both enzymes contributes to apparent mineralocorticoid excess. The insert shows the localization of the processes within the Henle loop. ACE: angiotensin converting enzyme, ENaC: epithelial sodium channel, ET-1: endothelin 1, HRE: hormone response element, NAD(H): nicotinamide adenine dinucleotide, NO: nitric oxide, ROMK: renal outer medullary potassium channel. Parts of the figure were drawn by using pictures from Servier Medical Art (http://smart.servier.com), licensed under a Creative Commons Attribution 3.0 Unported License (https://creativecommons.org/licenses/by/3.0).

Licorice intake induces physiological effects similar to aldosterone and corticosteroids. Resembling steroid-like structures, both 3MGA and GA are able to bind to the mineralocorticoid receptor (MR) in the distal tubules of the kidney (direct effect), although competitive binding assays revealed that the affinities of MR for licorice metabolites were up to 10,000 times weaker than those for adrenocortical hormones [49]. In a normal physiological state, MR is activated by aldosterone to increase sodium and water resorption into the blood and potassium excretion into the urine mediating sodium and water homeostasis within the kidneys. However, it is unclear how the direct effects of 3MGA and GA on MR contribute to the effect of licorice. Although there is some evidence of this direct effect in vitro [50], the relative affinity for MR compared to aldosterone as well as low serum levels of GA after licorice consumption, which did not reach the concentrations necessary to affect aldosterone or cortisol binding to MRs in humans, question that theory [51]. In addition, hyper-mineralocorticosteroid effects were not observed in patients or animals with severe adrenal insufficiency [52]. It is much more likely that metabolites of glycyrrhizin promote a change in cortisol metabolism [53]. Cortisol acts as an agonist for aldosterone to activate MR with equal affinity but circulates in 100–1000-times higher plasma concentrations than that of aldosterone. In adult tissues, the type II isozyme of 11β-hydroxysteroid dehydrogenase (11β-HSD2) is expressed in the distal nephron of the kidney [54], in smooth muscle cells and endothelial cells of the vascular wall [55], in the heart [56] and in the brain [57], where it serves to protect the MR from being overly activated by cortisol [53,58]. 11β-HSD2 converts ‘active’ cortisol to the ‘inactive’ cortisone which has a very low affinity for MR. Monder et al. [59] described a strong inhibitory effect of GA for 11β-HSD2 using rat kidney homogenates for in vitro analysis. In addition, oral glycyrrhizin administration inhibited renal 11β-HSD2 activity in rats in a dose-dependent manner [59,60]. Kato et al. [61] suggested that 3MGA, not GA, is the mainly causative agent of licorice-induced pseudohyperaldosteronism. In the kidneys, 11β-HSD2 inhibition by 3MGA or GA (K_i_: 5–10 nM) results in a significant increase of active cortisol concentration in the renal tissue leading to a syndrome of apparent mineralocorticoid excess (Figure 3a) [52,62]. In the vascular wall, it increases arterial tone enhancing contractile responses to pressor hormones and reducing endothelial nitric oxide production [57,63]. Further animal studies reported a markedly inhibitory effect of GA on hepatic ring A-reduction of aldosterone by two other hepatic enzymes (5β-reductase and 3β-hydroxysteroid dehydrogenase), increasing the circulating aldosterone levels [64].

A vasorelaxant effect of glabridin was described in rat mesenteric arteries, which was associated with the opening of potassium channels and a concomitant rise in tissue cyclic guanosine monophosphate levels [65].

Taken together, intake of licorice induces a mild form of apparent mineralocorticoid excess causing MRs to be activated by both cortisol and aldosterone via inhibition of enzymes necessary for their catabolism (Figure 3). The direct effects of 3MGA and GA on MRs seem to be only negligible in physiological conditions. In the kidney, MR activation leads to transcription of epithelial sodium channel (ENaC), Na^+^/K^+^ ATPase and mitochondrial enzymes, which accelerate adenosine triphosphate (ATP)-production (Figure 3b). The final consequences comprise elevated BP, sodium and water retention, decreased plasma potassium (hypokalemia) and caused a suppression of plasma renin and aldosterone levels [66]. In vascular smooth muscle cells, MR activation may further cause vascular stiffening by remodeling of the vascular wall [67]. Furthermore, direct effects of MR activation were described for the rat heart [68].

### 2.3. Licorice-Induced Hypertension

Licorice mediates its effect on BP primarily via the inhibition of renal 11β-HSD2 by 3MGA and GA (Figure 3a). Water and sodium retention in the kidney increase the blood volume and elevate BP [5]. The body countermeasures with a refractory lowering of the renin secretion in the kidneys, followed by decreased aldosterone production in the adrenal cortex via angiotensin II. However, the increasing level of cortisol (together with unrestricted activation of MR by cortisol) causes pseudohyperaldosteronism. This in turn results in further increasing blood volume and preload of the heart, thereby raising the mean arterial pressure. Furthermore, GA mediates the development of hypertension via decreased bioavailability of NO and activation of the vascular endothelin (ET-1) system (Figure 3a) which was accompanied by impaired endothelium-dependent relaxation in rats [69]. Activation of the endothelin system was also observed in human hypertension [70], and there is some evidence that increased ET-1 may be related to hypertensive end-organ damage and remodeling [71]. Interestingly, an infusion of GA into the rat brain elevated BP without affecting renal sodium and water resorption [72]. This finding indicated a central hypertensinogenic effect of licorice and suggested a more complex regulation of licorice-induced hypertension beyond the inhibition of 11β-HSD2.

Since a correlation between licorice ingestion and BP looks undeniable, further evaluation of quantities is necessary. Leskinen et al. [28] found that a daily intake of 290–370 mg licorice elevated both systolic and diastolic BP after two weeks. Furthermore, an increase of the extracellular fluid volume (hypervolemia) and amplified pressure wave reflection from the peripheral circulation was reported. Hautaniemi et al. [73] demonstrated that in addition to extracellular volume expansion, licorice increased stiffness of large arteries and systemic vascular resistance. A linear dose-response relationship between licorice intake and elevated BP was first proposed by Sigurjónsdóttir et al. [27], who found that a daily ingestion of 75 mg GA (~50 g of licorice) was sufficient to cause a significant increase in systolic BP within a period of two weeks. Similar correlations were later reported by a meta-analysis: analyzing the data of 18 studies (337 patients), systolic and diastolic BP seem to rise dose-dependently suggesting a public recommendation of avoiding excessive licorice consumption [74]. Based on the results of a 12-week experiment with 39 healthy female volunteers, van Gelderen et al. [75] proposed a no-effect level of 2 mg/kg GA per day (equal to 6 g licorice for a person with a body weight of 60 kg).

Two questions remain: 1. Is there any evidence that licorice will increase BP in patients dealing with hypotension? 2. Can general practitioners advocate the complementation of a normal diet with an intake of black licorice or other products containing GA in hypotensive patients? In 1994, it was reported that a 63-year old type 2 diabetic patient was treated for postural hypotension using licorice (3 g of GA/day) as treatment [76]. The patient’s BP increased from 90/60 mmHg to 130/80 mmHg in an upright position in 7 days of therapy. Thus, there might be some indications that licorice has its place in clinical therapy, but this must be further investigated in a double-blind, randomized, place-controlled trial to avoid bias.

The case reports of licorice-induced hypertension found in the literature range from mild and reversible forms to severe resistant hypertension requiring hospitalization. In consequence of the elevated BP some patients developed hypertensive encephalopathy or cerebrovascular accidents [77,78,79]. Acute heart failure, pulmonary edema [80,81,82] or generalized edema [83,84,85] can be caused by the sodium retaining effect of licorice (Figure 3a). Interestingly, the occurrence of edema associated with hypertension seems to be characteristic for the ‘licorice syndrome’. This is in contrast to true mineralocorticoid excess, where edema is typically absent as a result of the “sodium escape” phenomenon [86,87]. An observed increase in plasma concentration of atrial natriuretic peptide (ANP) after long-term consumption of licorice may be considered a physiological, albeit ineffective, response to prevent fluid retention and development of hypertension [88].

The effects of licorice on aldosterone secretion differ between the genders independently of the BP levels; women seem to be more susceptible to licorice intake [89,90]. A possible explanation for this gender difference are many other hormonal (estrogenic and antiandrogenic) effects exhibited by licorice in addition to its activity via MR. At least the alterations of the calcium metabolism that were observed in healthy women in response to licorice are probably influenced by several further components of the root such as glabridin, which shows estrogen-like activity [89].

There is very rare and controversial information about the correlation between licorice and the development of pulmonary hypertension. A possible contribution of licorice to pulmonary hypertension was suggested by Ruszymah et al. [91] after they had observed an increase in right atrial pressure and thickening of the pulmonary vessels of rats after GA administration. On the other hand, Yang et al. [92] described the attenuation of pulmonary hypertension progression and pulmonary vascular remodeling by glycyrrhizin in a monocrotaline-induced pulmonary hypertension rat model. Here, further studies are needed.

#### 2.3.1. Meta-analyses of Human Trials

In 2017, Penninkilampi et al. [74] reviewed the association between licorice intake, hypertension and hypokalemia. In a broad-based meta-analysis, they confirmed a significant increase in both systolic (5.45 mmHg; 95% confidence interval (CI) 3.51–7.39) and diastolic BP (3.19 mmHg; 95% CI 0.10–6.29) after chronic intake of products containing GA. Since physiological effects are not directly induced by licorice but rather by GA, the GA consumption was calculated for most of the studies. A GA content of 0.2% was approximated for black licorice [74] although the concentration of GA can obviously vary from product to product. Thus, the mean intake of 377.9 mg GA is equal to 189 g of licorice [74] and accounts for the described increase in systolic and diastolic BP. A further meta- and trial sequential analysis by Luis et al. [87] (26 trials, 985 patients) confirmed the significant increase in diastolic BP (1.74 mmHg; 95% CI 0.83–2.62) associated with the hypernatremia caused by licorice consumption. As mentioned by Penninkilampi et al. [74], most of the trials included in their meta-analysis were performed with volunteers. Selection bias in using volunteers and not random participants might be limiting results. The authors found that patients had higher increases in BP after a long intake of GA. They stratified the data in <4 weeks and ≥4 weeks and got elevations of 7.83 mmHg (95% CI 3.69–11.98) and 4.44 mmHg (95% CI 3.20–5.68), respectively. This confirmed the dose-response relationship and a positive correlation between GA dose and changes in both systolic and diastolic BP [74]. The significant increase of 5.45 mmHg might not cause adverse effects in a healthy individual. However, combined with hypokalemia, it can lead to problems in individuals dealing with uncontrolled hypertension [74]. There have been case reports of patients with hypertensive crises where high licorice-intake in combination with hypertension caused hospitalization [93]. Compared with the modest results found in the meta-analysis on the available literature, the number of case reports with serious events or death after chronic licorice ingestion appears excessive [74]. A history of high licorice consumption alone is mostly sufficient to induce a toxic state. The degree of hypokalemia can be severe to cause a lethal arrhythmia [5].

#### 2.3.2. Treatment

In most cases, hypertension and hypervolemia induced by licorice is reversible once intake is stopped. If treatment of licorice-induced hypertension should be necessary, patients will usually be treated as normal hypertensive patients with antihypertensive therapy [94]. Different biochemical analyses will indicate a state of hyperaldosteronism by displaying low plasma potassium and lower levels of plasma renin and aldosterone. Antihypertensive therapy that targets the MR, such as spironolactone, seems to be the primary choice [69]. In rats, it was shown that blocking MR normalized BP [69]. Spironolactone works as a competitive aldosterone antagonist reducing the number of ENaC and Na^+^/K^+^-ATPase in reverse to aldosterone and cortisol. However, spironolactone treatment is only suggested for an acute hypertensive crisis. Lifestyle interventions should be advised against chronic hypertension caused by high ingestion of licorice and GA-containing products. Depending on the severity, either less ingestion of licorice or a complete stop will be necessary. The ESC guidelines state that grade 2 or 3 hypertension have to be treated with antihypertensive therapy [32]. This accounts for a clinically measured systolic BP ≥ 160 mmHg and/or a diastolic BP ≥ 100 mmHg. Since the effects on electrolyte-levels are delayed, it is furthermore important to stabilize electrolytes, with specific focus of on potassium. When licorice-induced hypertension is treated, it should be kept in mind that it can take up to six months to reverse the mineralocorticoid-like effects of licorice due to its long half-life and the duration required to normalize the renin-angiotensin-aldosterone-system [95].

Indeed, the ESC guidelines for treating hypertension mention that the intake of licorice could influence BP. They address that the medical history should include use of licorice [32]. However, there are no further comments on how licorice-induced hypertension should be treated. An intervention study aimed to investigate whether hypertensive patients were more sensitive to the inhibition of 11β-HSD2 than normotensive patients [96] and found that after 4 weeks of licorice consumption, the mean increase in systolic BP was 3.5 mmHg in healthy individuals and 15.3 mmHg in hypertensive subjects. The mean rise in diastolic BP confirmed this with an increase of 3.6 in mmHg in normotensive and 9.3 mmHg in hypertensive patients. The *p*-value showed significant differences in both systolic (*p* = 0.004) and diastolic BP (*p* = 0.03) [96]. Thus, the authors concluded that subjects with essential hypertension are more sensitive to the licorice-induced inhibition of 11β-HSD2 than normotensive subjects. This finding supports the suggestion that licorice might have stronger adverse effects in patients suffering from hypertension.

However, the available data on this topic is limited and of modest quality and only one clinical trial can be found (Table 1). Further double-blind randomized placebo-controlled studies would be necessary to determine the clinical effects of licorice intake in both healthy and non-healthy individuals.

### 2.4. Cardiovascular Effects of Licorice

Licorice traditionally has been prescribed for treatment of cardiovascular disorders, but its effects are not just benign. From the cardiovascular complication described in the literature, cardiac arrhythmias are the most serious side effect caused by licorice intake due to severe hypokalemia (Figure 3a) [105]. The depletion of the body’s potassium stores can cause a prolongation of the QT interval, which is closely connected with ventricular arrhythmias and tachycardia [106]. As a consequence, several patients experienced a cardiac arrest with a subsequent recovery [107,108,109]. Konik et al. [110] described a case of coronary artery spasm induced by licorice. The vasospastic effect of licorice was attributed to changes in endothelin and nitric oxide systems. Recently, a Polish clinical study found a correlation of arterial stiffness parameters with estimated cardiovascular risks in humans [111]. Transient visual loss, migraines and posterior reversible encephalopathy syndrome has also been demonstrated in a few cases. It is assumed that GA inhibits angiogenesis due to inhibition of reactive oxygen species generation [112]. Sobieszczyk et al. [102] found an additional attenuated vascular smooth muscle vasodilatory function without BP changes in healthy humans after 11β-HSD2 inhibition through GA. They proposed that in states of 11β-HSD2 inactivation, non-aldosterone-mediated activation of vascular MRs may contribute to vascular dysfunction and possibly to CVDs.

In rats, cardioprotective effects of licorice and its metabolites were observed, which are mostly related to their antioxidant properties. Thirty days of licorice intake improved cardiac function and preserved histology of cardiomyocytes either by augmentation of endogenous antioxidants or by reduction in oxidative stress. Thus, licorice may delay the progression of ischemic heart disease [113]. Ohja et al. [114] further described a cardioprotective effect against oxidative stress in myocardial ischemia-reperfusion injury after ingestion of *Glycyrrhiza glabra*. Another animal study indicated that GA protects against isoproterenol-induced oxidative stress in rat myocardium decreasing lipid hydroperoxides and isoprostanes and increasing superoxide dismutase and glutathione levels [115].

Some studies suggested that the flavonoid glabridin may also have beneficial effects on the cardiovascular system. The effects described comprise inhibition of low density lipoprotein oxidation and atherogenesis [116], a possible inhibition of NADPH oxidase or an increase in the expression of antioxidant enzymes observed in macrophages [117]. Glabridin also stimulates DNA synthesis in human endothelial cells and demonstrated a bi-phasic proliferative effect on human vascular smooth muscle cells. The combination of an inhibition of smooth muscle cell proliferation and an induction of endothelial cell proliferation may be beneficial for the prevention of atherosclerosis [118,119]. Most recently, Huang et al. [120] reported that glabridin is able to prevent doxycyclin-induced cardiotoxicity in mice through the prevention of gut microbiota dysbiosis. Nevertheless, it remains unclear to which extent these effects contribute to the putative therapeutic actions of licorice.

### 2.5. Interaction of Licorice with Prescribed Drugs

Licorice can interfere with cardiac medications, e.g., with drugs used in the treatment of hypertension such as angiotensin converting enzyme (ACE)-inhibitors [121]. Some licorice compounds including glabridin and GA can interact with other drugs and the human liver microsomal cytochromes P450 and P450 3A4 (CYP3A4) systems [122,123,124,125]. Animal [126,127] and human studies [103] showed that glycyrrhizin has an inductive effect on CYP3A including CYP3A4 and the effect on CYP3A4 may be related to an activation of human pregnane X receptor (hPXR) [103,128]. Other studies described that CYP3A4 was inactivated by licorice extract and glabridin in a time- and concentration-dependent manner [124]. CYP3A4 is involved in the metabolism of xenobiotics [122], roughly half the drugs that are in use today, suggesting that the influence of licorice on CYP3A4 activity needs to be further investigated.

Heck et al. [129] described a toxic effect potentiation of warfarin, a cardiac drug that requires strict dosage adjustment, due to the inhibition of the hepatic microsomal enzymes by licorice.

Matsumoto et al. [130] investigated the effects of licorice on ABC-transporters. Using an in vivo ATPase assay, they demonstrated that licorice root and GA can inhibit P-glycoprotein. A two-phase randomized crossover trial by Yan et al. [104] revealed at least no induction effect on the P-glycoprotein expression after continuous glycyrrhizin administration (225 mg/day) for 6 days. The authors proposed that further research was needed to study the direct inhibition effect of glycyrrhizin on P-glycoprotein. For the pharmaceutical use, it is important to know and consider the interaction between licorice and drugs metabolized by CYP3A4 and P-glycoprotein.

Licorice decreases the bioavailability of cyclosporine and is thus contraindicated [120] in conjunction with this drug [128]. The intake of licorice should be done with caution, when using antihypertensive drugs. ACE-inhibitors, e.g., captopril, inhibit the angiotensin converting enzyme, limiting levels of angiotensin and aldosterone. It was shown that ACE-inhibitors enhance the effects of 11β-HSD2 which may contribute to the natriuretic effect [131]. Hypokalemia is one of the most serious adverse effects of licorice intake and should be completely avoided with loop-diuretics and thiazides since it can lead to serious hypokalemia and hospitalization [132].

The combination of medicine containing licorice and digitalis can cause toxicity, especially in elderly patients. There has been one reported case of digoxin toxicity due to licorice-induced hypokalemia [133].

### 2.6. Contraindications and Effects of Licorice Overconsumption

Licorice and its derivatives are affirmed as ‘Generally Recognized as Safe’ (GRAS) for use in foods by the United States Food and Drug Administration (21 CFR 184.1408). Nevertheless, tolerable upper limits of licorice intake have been provided by several institutions: the European Scientific committee of Food recommends that the daily ingestion should not exceed 100 mg of glycyrrhizin (60–70 g of licorice) [134]; the Dutch Nutrition Information Bureau advised against a daily consumption above 200 mg of glycyrrhizin (150 g of licorice) [2]. Since most consumers are not aware of possible health hazards, and there are currently no precise declaration data of glycyrrhizin on food, it is difficult to control licorice intake. Furthermore, it has to be investigated if sporadic intake carries the same risks compared to the daily consumption that is analyzed in most studies. Licorice is found in diet gum, cough mixtures, tea and herbal medicine. Having a mixed intake of these products will accumulate the quantity of GA in vivo, and therefore, increase the risk of symptoms.

In general, people aged over 40, patients with history of cardiac disease or more susceptible to cardiac arrhythmias should avoid excess licorice intake in order to obviate arrhythmias or cardiac arrest caused by licorice-induced hypokalemia. One study investigated patients treated with traditional Japanese medicine containing licorice [135]. They discovered that 24.2% of the patients treated with this medicine developed hypokalemia 34 days after administration. Hypokalemia is a serious state that increases the risk of arrhythmia and is associated with an up to 10-time increase in all-cause mortality [74]. The meta-analysis by Penninkilampi et al. [74] summarized other side effects including rhabdomyolysis, paralysis, hypertensive encephalopathy and cardiac arrest. That is why patients who are on medicines lowering potassium levels (such as thiazide or loop diuretics) should also minimize their licorice intake. The same applies for patients suffering from diarrhea or alcoholism, which can worsen hypokalemia. Licorice can be dangerous in patients treated with antihypertensive drugs such as ACE-inhibitors and diuretics. Due to the salt-retaining effect of 3MGA and GA, people suffering from congestive heart failure or resistant hypertension should completely abstain from products containing licorice. This is also advisable for patients taking digoxin or warfarin to avoid the risk of toxicity. Since 3MGA and GA are known to inhibit 11β-HSD2, licorice ingestion during pregnancy should be avoided. GA consumption impaired the development of the respiratory systems in rats because the conversion of cortisone into cortisol plays an important role in lung maturation [136].

## 3. Conclusions

In recent years, the mechanisms of action of licorice and its active components have become understood in more detail. The use of licorice in herbal medicine is obviously a result of some positive effects. Hence, it has become one of the most used herbs in traditional Chinese medicine and is still used in China to treat gastric symptoms and respiratory diseases today [10]. Numerous studies have reported about effects of the different compounds found in the licorice root. Glabridin has been proven to be a potent antioxidant with hypoglycemic effects [21]. Referring to studies, glycyrrhizin possesses a wide range of pharmacological effects described as antiulcer and anti-inflammatory [11,12,13], antiviral [14,15], anticariogenic [137,138] and antispasmodic [139,140].

The utilization of some licorice compounds in a clinical setting is still under investigation. This applies also for artificial GA derivatives such as carbenoxolone [141] or acetoxolone [142]. Glycyrrhizin was identified as an attractive drug candidate for cancer therapy after demonstrating an apoptotic effect on tumor cells [143]. Today, researchers are intensely investigating the applicability of licorice in treatment of breast and prostate cancer. The antitumor activity has attracted the attention of many scientists, since cancer is still one of the leading causes of death in humans around the globe [10].

Nevertheless, due to some safety considerations associated with chronic high-dose intake, licorice should still be consumed with caution. With the elucidation of licorice constituents and the discovery that 3MGA and GA affect the renin-angiotensin-aldosterone-system, pseudohyperaldosteronism is the obvious adverse effect; however, other side effects such as hypertension, hypokalemia and hypernatremia have also been proven. If left untreated, they can cause arrhythmia and, in a worst-case scenario, cardiac arrest. Omar et al. [30,40] have described in detail why licorice should be handled more as medicine than as a candy and that excess licorice consumption can cause serious life-threatening complications, especially in individuals already dealing with high BP and patients under treatment with anti-hypertensive drugs. Adverse effects of high-dose licorice intake have been attributed to glycyrrhizin, 3MGA and GA. Since the final toxicology report has been published in 2007, therapeutic doses of licorice are generally recommended as safe in humans [144]. Especially Scandinavian countries have a higher intake of licorice, and in addition, their licorice has a higher GA concentration [121]. This suggests a need of public focus on the negative effects of licorice on cardiovascular health. However, this is also needed in China, where licorice is widely used in medical practice; here, the knowledge of licorice’s interaction with prescription medicines is quite important to avoid possible iatrogenic accidents.

## Figures and Tables

**Table 1:** Studies investigating the effects of licorice intake on the human cardiovascular system.

Author (Year), Country	Study Design	*n*	Drug	Daily Dose	Duration	Relevant Results
Epstein et al. (1977) [97], New Zealand	Pre-post intervention	14	Licorice	100–200 g	1–4 weeks	Serious metabolic effects due to modest licorice intake.
Forslund et al. (1989) [88], Finland	Pre-post intervention	15	Licorice	100 g	8 weeks	Increase in plasma ANP; Decrease in antidiuretic hormone, aldosterone, and plasma renin activity.
MacKenzie et al. (1990) [98], The Netherlands	Pre-post intervention	10	GA	500 mg	8 days	Inhibition of 11β-HSD2.
Kageyama et al. (1992) [99], Japan	Pre-post intervention	58	Glycyrrhizin	225 mg	7 days	Changes in cortisol metabolism.
Bernadini (1994) [100], Italy	Pre-post intervention		Licorice root extract	108-814 mg glycyrrhizin	14 days	Depression of plasma renin activity favored by subclinical disease.
Armanini et al. (1996) [101], Italy	Pre-post intervention	6	Licorice concentrate	7 g (500 mg GA)	7 days	Decreased activity of 11β-HSD2.
van Gelderen et al. (2000) [75], USA	Double-blind randomized controlled	39	GA	0–4 mg per kg	8 weeks	No-effect level: 2 mg/kg GA per day.
Sigurjónsdóttir et al. (2001) [27], Iceland/Sweden	Pre-post intervention	24	Licorice	50–200 g	2–4 weeks	Increase in SBP.
Sigurjónsdóttir et al. (2003) [96], Sweden	Pre-post intervention	25	Licorice	100 g	4 weeks	Increase in SBP and DBP. Subjects with essential hypertension are more sensitive to licorice-induced rise in BP.
Sigurjónsdóttir et al. (2006) [90], Sweden	Pre-post intervention	25	Licorice	100 g	4 weeks	The effect on aldosterone secretion differs between the genders.
Sobieszcyk et al. (2010) [102], USA	Randomized double-blind placebo-controlled crossover	15	GA	130 mg	14 days	Attenuated vasodilatory function on VSMCs.
Tu et al. (2010) [103], China	Two-phase randomized crossover	16	Glycyrrhizin	2 × 150 mg	14 days	Induction of CYP3A.
Yan et al. (2013) [104], China	Two-phase randomized crossover	14	Glycyrrhizin (salt tablet)	3 × 75 mg	6 days	No induction of P-glycoprotein.
Leksinen et al. (2014) [28], Finland ClinicalTrials: NCT01742702	Non-randomized, controlled open label	20	Licorice	290–370 mg glycyrrhizin	14 days	Increase in SBP, DBP, extracellular volume and amplified pressure wave reflection from the periphery.
Hautaniemi et al. (2017) [73], Finland	Non-randomized, controlled open label	22	Licorice	290–370 mg glycyrrhizin	14 days	Increase in SBP, DBP, central pulse pressure, extracellular fluid volume and aortic to popliteal pulse wave velocity.

11β-HSD2: 11-β-hydrogenase type II enzyme; ANP: atrial natriuretic peptide; BP: blood pressure; CYP3A: cytochrome P450 3A4; DBP: diastolic blood pressure; GA: 18β-glycyrrhetinic acid; SBP: systolic blood pressure; VSMC: vascular smooth muscle cell.

## Abbreviations

11β-HSD2	11-β-hydrogenase type II enzyme
3MGA	3β-monoglucuronyl-18β-glycyrrhetinic acid
ACE	Angiotensin converting enzyme
ANP	Atrial natriuretic peptide
ATP	Adenosine triphosphate
BP	Blood pressure
CI	Confidence interval
CVD	Cardiovascular disease
CYP3A4	Cytochrome P450 3A4
DBP	Diastolic blood pressure
ENaC	Epithelial sodium channel
ESC	European Society of Cardiology
ET-1	Endothelin 1
GA	18β-glycyrrhetinic acid
HRE	Hormone response element
MR	Mineralocorticoid receptor
NAD(H)	Nicotinamide adenine dinucleotide
NO	Nitric oxide
ROMK	Renal outer medullary potassium channel
SBP	Systolic blood pressure
VSMC	Vascular smooth muscle cell
